# Solid-State Nonlinear Optical Properties of Mononuclear Copper(II) Complexes with Chiral Tridentate and Tetradentate Schiff Base Ligands

**DOI:** 10.3390/ma12213595

**Published:** 2019-11-01

**Authors:** Luca Rigamonti, Alessandra Forni, Elena Cariati, Gianluca Malavasi, Alessandro Pasini

**Affiliations:** 1 Dipartimento di Scienze Chimiche e Geologiche, Università degli Studi di Modena e Reggio Emilia, via G. Campi 103, 41125 Modena, Italy; gianluca.malavasi@unimore.it; 2 Dipartimento di Chimica, Università degli Studi di Milano, via C. Golgi 19, 20133 Milano, Italy; alessandra.forni@istm.cnr.it (A.F.); elena.cariati@unimi.it (E.C.); alessandro.pasini@unimi.it (A.P.); 3 Istituto di Scienze e Tecnologie Molecolari, Consiglio Nazionale delle Ricerche, via C. Golgi 19, 20133 Milano, Italy

**Keywords:** chiral diamines, template synthesis, mononuclear copper(II) complexes, Schiff base ligands, nonlinear optics, Kurtz–Perry powder technique

## Abstract

Salen-type metal complexes have been actively studied for their nonlinear optical (NLO) properties, and push-pull compounds with charge asymmetry generated by electron releasing and withdrawing groups have shown promising results. As a continuation of our research in this field and aiming at solid-state features, herein we report on the synthesis of mononuclear copper(II) derivatives bearing either tridentate N_2_O Schiff bases L^(a−c)−^ and pyridine as the forth ancillary ligand, [Cu(L^a−c^)(py)](ClO_4_) (**1a**–**c**), or unsymmetrically-substituted push-pull tetradentate N_2_O_2_ Schiff base ligands, [Cu(5-A-5′-D-saldpen/chxn)] (**2a**–**c**), both derived from 5-substituted salicylaldehydes (sal) and the diamines (1*R*,2*R*)-1,2-diphenylethanediamine (dpen) and (1*S*,2*S*)-1,2-diaminocyclohexane (chxn). All compounds were characterized through elemental analysis, infrared and UV/visible spectroscopies, and mass spectrometry in order to guarantee their purity and assess their charge transfer properties. The structures of **1a**–**c** were determined via single-crystal X-ray diffraction studies. The geometries of cations of **1a**–**c** and of molecules **2a**–**c** were optimized through DFT calculations. The solid-state NLO behavior was measured by the Kurtz–Perry powder technique @1.907 µm. All chiral derivatives possess non-zero quadratic electric susceptibility (*χ*^(2)^) and an efficiency of about 0.15–0.45 times that of standard urea.

## 1. Introduction

Since the generation of optical harmonics by the interactions of a ruby optical maser [1] with crystalline quartz [2], the research in the nonlinear optics (NLO) field [3] has grown exponentially [4,5,6,7,8,9]. One of the most extensively applied features of NLO materials is the generation of second harmonic (SHG) radiation, that is, the process where the frequency *ω* of an incident radiation becomes 2*ω* when passing through the medium [3]. Molecular compounds able to show and maximize such an NLO effect in the solid state have to possess high molecular quadratic hyperpolarizabily (*β*) values together with a non-centrosymmetric crystal packing [10], able to guarantee the final non-zero quadratic electric susceptibility (*χ*^(2)^) of the bulk material [7,8].

Among other organometallic [11,12,13,14,15] and coordination compounds [14,15,16,17,18], salen-type [19] metal complexes have been actively studied for their NLO properties, mostly for the last two decades [17,18,20,21,22,23]. The best functionalization of these derivatives has been achieved by inserting donor (D) groups on the salicylaldehyde (sal) moieties and acceptor (A) groups on the diamine bridge, such as substituted 1,2-diaminobenzene (phen) and diaminomaleonitrile (damn). Such push-pull bis(salicylaldiminato) metal complexes with charge asymmetry generated by electron releasing and withdrawing groups on the ligand skeleton have shown promising results for their SHG efficiency in solution through electric field induced second harmonic (EFISH) generation and hyper rayleigh scattering (HRS) measurements [24,25,26,27,28,29,30,31,32,33]. This class of compounds has also been the subject of extensive theoretical studies through the years in order to elucidate their electronic and optical features [17,26,32,34,35,36,37]. As an alternative to the above-mentioned push-pull structure, we focused our attention on salen-type metal complexes in which the A–D asymmetry is achieved by push-pull unsymmetrical substitution on the sal moieties, firstly on copper(II) derivatives [38], and very recently also on nickel(II) compounds [39]. Other examples have also applied such an approach with different designs and synthetic strategies [21,40,41,42,43,44].

After the first evidences of the role of salen-type complexes in solid-state NLO measurements [45,46] and the promising results in solution [24,25,26,27], the first real attempt to engineer this class of compounds for solid-state applications shortly arrived by Lacroix et al., by introducing the chiral diamine 1,2-diaminocyclohexane (chxn) in the complexes [Ni(4,4′-diNEt_2_-sal-(1*R*,2*R*)-chxn)]∙EtOH and [Mn(Cl)(4,4′-diNEt_2_-sal-(1*R*,2*R*)-chxn)], which ensures the crystallization of the two compounds in the non-centrosymmetric monoclinic *P*2_1_ and orthorhombic *P*2_1_2_1_2_1_ space groups, respectively. Their bulk NLO response was measured by the Kurtz–Perry technique [47], and their powder SHG efficiencies were <0.25 and 8 @1.907 µm and 0.1 and 0.5 @1.064 µm that of standard urea, respectively. The reduction of the efficiencies @1.064 µm was explained by absorption phenomena [23,48]. The derivative [Ni(4,4′-diNEt_2_-sal-(1*R*,2*R*)-dpen)] with 1,2-diphenylethylenediamine (dpen) was then synthesized by changing the chemical nature of the chiral diamine, and studied for its solid-state NLO response. The powder SHG efficiency @1.907 µm of this nickel(II) complex, which crystallizes in the monoclinic *P*2_1_ space group, is 13 times that of urea [23,49], close to the upper limit [10] in this class of complexes [17]. As a reference, 3-methyl-4-nitropyridine-1-oxide (POM), a molecular material that entered the market also thanks to its transparency and ability to be grown as large crystals, has a large SHG efficiency equal to 13 times that of urea [50].

**Scheme 1:**
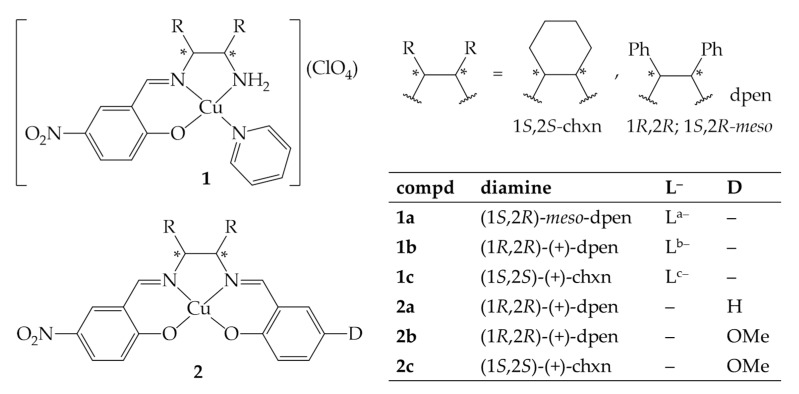
Schemes of compounds [Cu(L^a–c^)(py)](ClO_4_) (**1a**–**c**) and [Cu(5-A-5′-D-saldpen/chxn)] (**2a**–**c**), the object of this work.

Salen-type copper(II) complexes with chiral diamines have also been known for several decades [51,52,53,54,55,56]. They have been studied in particular for catalysis [56,57,58,59,60] and, in some cases, for biological applications [61,62,63]. Among them, there are also two examples studied for their NLO properties, a homodinuclear Cu_2_ compound, which unfortunately crystallizes in a centrosymmetric space group [64], and a heterodinuclear CuGd complex, whose SHG efficiency reaches 0.3 times that of urea [65]. Nevertheless, to the best of our knowledge, there are no examples of chiral mononuclear copper(II) compounds studied for their solid-state NLO features. In this work, we extend our previous studies on unsymmetrically-substituted salen derivatives [38] using chiral diamines, dpen and chxn, as a bridge between the imine nitrogen donor atoms (see Scheme 1) in the substitution of the aliphatic ethylenediamine (en) and 1,3-diaminopropane (tn).

Both dpen and chxn exist in three stereoisomers, (1*R*,2*R*) and (1*S*,2*S*) enantiomers and (1*S*,2*R*) mesomeric form. In the light of all the multiple combinations of possible A/D groups and chirality given by the diamines, we decided to focus our efforts on representative compounds, first of all by keeping NO_2_ as the acceptor group, thanks to its fundamental role in both the enhancement of the molecular NLO response *β* and the intensity of the ligand-to-metal charge transfer (LMCT) band [38]. We then selected (1*R*,2*R*)-(+)-dpen and (1*S*,2*S*)-(+)-chxn as diamines, and the insertion of the OMe group was evaluated for its cooperative effect with NO_2_. The synthetic intermediates [Cu(L^a−c^)(py)](ClO_4_) (**1a**–**c**), with tridentate N_2_O Schiff base ligands L^(a–c)−^ and pyridine as the forth ancillary ligand, and the final copper(II) complexes [Cu(5-A-5′-D-saldpen/chxn)] (**2a**–**c**), with unsymmetrically-substituted tetradentate N_2_O_2_ Schiff base ligands, are reported in Scheme 1. Furthermore, both final push-pull compounds **2a**–**c** and intermediates **1a**–**c** were tested for their solid-state NLO response by measuring their SHG efficiency through the Kurtz–Perry technique.

## 2. Materials and Methods

### 2.1. General Information

All chemicals were reagent grade purchased from Sigma Aldrich (purity ≥ 97%) and solvents (methanol, MeOH; diisopropyl ether, *i*Pr_2_O) were used as received. Elemental analyses were performed at the Mycroanalytical Laboratory of the Università degli Studi di Milano. Electrospray ionization mass spectrometry (ESI-MS) experiments were performed with an LCQ Advantage Thermofluxional Instrument (Thermo Scientific, Waltham, USA) on MeOH solutions of the compounds (Figure S1 in Supplementary Materials, SM). Infrared (IR) spectra were recorded as KBr disks using a Jasco FT-IR 410 spectrophotometer (Jasco, Tokyo, Japan) with a 2 cm^−1^ resolution (Figure S2 in SM). UV/visible spectra were recorded at 298 K with a Jasco V-570 UV/Vis/NIR spectrophotometer (Jasco, Tokyo, Japan) both in reflectance (solid samples) and in transmittance (solution samples) modes in the 200–900 nm range; solution studies were performed on **1b** and **1c** from 10^−3^ to 4 × 10^−5^ mol L^−1^ MeOH solutions and then by addition of increasing pyridine amounts to the 4 × 10^−5^ mol L^−1^ MeOH solutions, and on **2a**–**c** from 10^−3^ to 2 × 10^−5^ mol L^−1^ CHCl_3_ solutions; *λ* values are accurate to ±1 nm and spectra are reported as wavenumbers, in cm^−1^ (*ε*, L mol^−1^ cm^−1^).

### 2.2. Synthesis of [Cu(L^a^)(py)](ClO_4_) (**1a**)

An aqueous solution (2 mL) of Cu(ClO_4_)_2_ 6H_2_O (250 mg, 0.67 mmol) and py (162 µL, 2.01 mmol) was added to a stirred solution of 5-NO_2_-salH (112 mg, 0.67 mmol) in MeOH (7 mL). The mixture was stirred at room temperature for 1 h when (1*S*,2*R*)-*meso*-dpen (141 mg, 0.67 mmol) was added; after 3 h under stirring at room temperature, the dark solution was left aside to slow evaporation for three days until the title compound was precipitated as black needles (suitable for X-ray diffraction), filtered, washed with MeOH and *i*Pr_2_O, and dried under vacuum (260 mg, 65%). ESI-MS (MeOH): *m/z* 423 ([Cu(L^a^)]^+^, 70%), 455 ([Cu(L^a^)(MeOH)]^+^, 75), 845 ([Cu_2_(L^a^)_2_−H]^+^, 100). Elemental analysis calcd (%) for C_26_H_23_N_4_O_7_CuCl·MeOH∙½H_2_O (643.54): C, 50.39; H, 4.39; N, 8.71. Found: C, 50.30; H, 3.85; N, 8.60. FT-IR (KBr): *ν*(NH_2_) 3319, 3267, *ν*(C=N) 1634, 1607, *ν*(NO_2_) 1315, *ν*(ClO_4_) 1103 cm^−1^.

### 2.3. Synthesis of [Cu(L^b^)(py)](ClO_4_) (**1b**) and Isolation of [Cu(L^b^)(H_2_O)](ClO_4_) (**1b’**) as Intermediate

An aqueous solution (2.5 mL) of Cu(ClO_4_)_2_ 6H_2_O (360.0 mg, 0.97 mmol) and py (235 μL, 2.91 mmol) was added to a stirred solution of 5-NO_2_-salH (162.0 mg, 0.97 mmol) in MeOH (10 mL). The mixture was stirred at room temperature for 1 h when (1*R*,2*R*)-(+)-dpen (206.0 mg, 0.97 mmol) was added with the concomitant precipitation of **1b’** as a violet solid, which was collected after 3 h under stirring, washed with MeOH and *i*Pr_2_O, and dried under vacuum (275.0 mg, 52%). ESI-MS (MeOH): *m/z* 441 ([Cu(L^b^)(H_2_O)]^+^, 100%). Elemental analysis calcd (%) for C_21_H_20_ClCuN_3_O_7_∙½H_2_O (550.41): C, 45.83; H, 3.85; N, 7.63. Found: C, 45.80; H, 4.23; N, 7.52. FT-IR (KBr): *ν*(NH_2_) 3315, 3259, *ν*(H_2_O) 3296, *ν*(C=N) 1626, 1604, *ν*(NO_2_) 1333, *ν*(ClO_4_) 1082 cm^−1^.

Starting from an aqueous solution (3 mL) of Cu(ClO_4_)_2_ 6H_2_O (440.0 mg, 1.20 mmol), py (290 μL, 3.60 mmol), 5-NO_2_-salH (200.0 mg, 1.20 mmol) in MeOH (15 mL), and (1*R*,2*R*)-(+)-dpen (255.0 mg, 1.20 mmol), after 3 h under stirring, an excess of pyridine (1 mL) was added and the mixture was further stirred at room temperature for two days, until all **1b’** disappeared. The green solution was left aside to slow evaporation for one week, yielding **1b** as a dark green solid that was filtered, washed with MeOH and *i*Pr_2_O, and dried under vacuum (540 mg, 75%). ESI-MS (MeOH): *m/z* 455 ([Cu(L^b^)(MeOH)]^+^, 60%), 909 ([Cu_2_(L^b^)_2_(MeOH)_2_−H]^+^, 100). Elemental analysis calcd (%) for C_26_H_23_ClCuN_4_O_7_ (602.47): C, 51.83; H, 3.85; N, 9.30. Found: C, 51.80; H, 4.30; N, 9.01. FT-IR (KBr): *ν*(NH_2_) 3318, 3269, *ν*(C=N) 1645, 1608, *ν*(NO_2_) 1315, *ν*(ClO_4_) 1098 cm^−1^.

### 2.4. Synthesis of [Cu(L^c^)(py)](ClO_4_) (**1c**)

An aqueous solution (2 mL) of Cu(ClO_4_)_2_ 6H_2_O (385.4 mg, 1.05 mmol) and py (170 μL, 2.10 mmol) was added to a stirred solution of 5-NO_2_-salH (175.2 mg, 1.05 mmol) in MeOH (10 mL). The mixture was stirred at room temperature for 1 h when (1*S*,2*S*)-(+)-chxn (118.0 mg, 1.05 mmol) was added, and the mixture was left under stirring for 3 h. The title compound was collected as a violet solid by filtration, washed with MeOH and *i*Pr_2_O, and dried under vacuum (320.5 mg, 63%). ESI-MS (MeOH): *m/z* 325 ([Cu(L^c^)]^+^, 20%), 357 ([Cu(L^c^)(MeOH)]^+^, 100). Elemental analysis calcd (%) for C_18_H_21_ClCuN_4_O_7_ (504.39): C, 42.86; H, 4.20; N, 11.11. Found: C, 42.97; H, 4.40; N, 10.90. FT-IR (KBr): *ν*(NH_2_) 3300, 3248, *ν*(C=N) 1647, 1604, *ν*(NO_2_) 1315, *ν*(ClO_4_) 1104 cm^−1^.

### 2.5. Synthesis of [Cu(5-NO_2_-5′-H-sal-(1R,2R)-dpen] (**2a**)

salH (37.6 mg, 0.31 mmol) and NaOH (0.45 mL of a 0.64 mol L^−1^ aqueous solution, 0.29 mmol) were added to a solution of **1b** (142.4 mg, 0.24 mmol) in MeOH (10 mL), and the reaction mixture was refluxed for 1 h. The resulting violet solid was filtered, washed with MeOH and *i*Pr_2_O, and dried under vacuum (95.1 mg, 77%). ESI-MS (MeOH): *m/z* 527 ([M + 1]^+^, 10%), 1053 ([2M + 1]^+^, 35), 1075 ([2M + Na]^+^, 100). Elemental analysis calcd (%) for C_28_H_21_CuN_3_O_4_ (527.04): C, 63.81; H, 4.02; N, 7.97. Found: C, 63.94; H, 4.41; N, 8.02. FT-IR (KBr): *ν*(C=N) 1632, 1604, *ν*(NO_2_) 1314 cm^−1^.

### 2.6. Synthesis of [Cu(5-NO_2_-5′-OMe-sal-(1R,2R)-dpen] (**2b**)

5-OMe-salH (41.0 mg, 0.27 mmol) and NaOH (0.35 mL of a 0.76 mol L^−1^ aqueous solution, 0.27 mmol) were added to a solution of **1b** (133.2 mg, 0.22 mmol) in MeOH (10 mL), and the reaction mixture was refluxed for 30 min. The resulting military green solid was filtered, washed with MeOH and *i*Pr_2_O, and dried under vacuum (110.2 mg, 92%). ESI-MS (MeOH): *m/z* 557 ([M + 1]^+^, 25%), 1113 ([2M + 1]^+^, 60), 1135 ([2M + Na]^+^, 100). Elemental analysis calcd (%) for C_29_H_23_CuN_3_O_5_ (557.06): C, 62.53; H, 4.16; N, 7.54. Found: C, 62.56; H, 4.67; N, 7.28. FT-IR (KBr): *ν*(C=N) 1634, 1603, *ν*(NO_2_) 1313 cm^−1^.

### 2.7. Synthesis of [Cu(5-NO_2_-5′-OMe-sal-(1S,2S)-chxn] (**2c**)

5-OMe-salH (46.2 mg, 0.30 mmol) and NaOH (0.40 mL of a 0.76 mol L^−1^ aqueous solution, 0.30 mmol) were added to a solution of **1c** (128.3 mg, 0.26 mmol) in MeOH (10 mL), and the reaction mixture was refluxed for 30 min. The resulting dark green solid was filtered, washed with MeOH and *i*Pr_2_O, and dried under vacuum (70.1 mg, 59%). ESI-MS (MeOH): *m/z* 459 ([M + 1]^+^, 55%), 481 ([M + Na]^+^, 15), 939 ([2M + Na]^+^, 100). Elemental analysis calcd (%) for C_21_H_21_CuN_3_O_5_ (458.96): C, 54.96; H, 4.61; N, 9.16. Found: C, 54.57; H, 5.02; N, 8.84. FT-IR (KBr): *ν*(C=N) 1635, 1600, *ν*(NO_2_) 1316 cm^−1^.

### 2.8. X-ray Data Collection and Structure Determination

Crystals suitable for X-ray diffraction (XRD) experiments were obtained directly by slow evaporation of the MeOH reaction mixture for **1a** and **1b**, and by diffusion of *n*-hexane into an acetone solution for **1c**. Crystal data and details of data collection are summarized in Table 1. Intensity data for **1a** were collected on the XRD1 beamline of the Elettra Synchrotron Light Laboratory (Trieste, Italy), using radiation *λ* = 0.8 Å, owing to the very small dimensions of the crystals. For **1b** and **1c**, data collection was performed on a Bruker Apex II diffractometer (Bruker AXS Inc., Madison, WI, USA) using graphite monochromatic Mo–K*α* radiation (*λ* = 0.71073 Å).

During data collections, no crystal decay was observed, so no time-decay correction was needed. Data reductions were performed with MOSFLM version 6.11c [66] and SCALA [67] (for **1a**) and with SAINT and SADABS [68] (for **1b** and **1c**). All the structures were solved by direct methods and refined with SHELXL-2016/6 [69] implemented in WinGX–Version 2014.1 system [70]. The program Mercury was used for graphics [71]. Anisotropic thermal parameters were used for all non-hydrogen atoms. The isotropic thermal parameters of H atoms were fixed at 1.2 (1.5 for methylene groups) times those of the atom to which they were attached. All H atoms were placed in calculated positions and refined by a riding model.

CCDC 1954460-1954462 contains the supplementary crystallographic data for **1a**–**c**, respectively. These data can be obtained free of charge via http://www.ccdc.cam.ac.uk/conts/retrieving.html, or from the Cambridge Crystallographic Data Centre, 12 Union Road, Cambridge CB2 1EZ, UK; fax: (+44) 1223-336-033, or e-mail: deposit@ccdc.cam.ac.uk.

### 2.9. Computational Details

Geometry optimizations of the cations [Cu(L^a–c^)(py)]^+^ of **1a**–**c** and of **2a**–**c** were performed at UM06/6-311++G(d,p) level of theory. For **1a**–**c**, the respective single crystal X-ray structures were used as a starting point, while for **2a**–**c**, the initial guess for optimization was build up from the optimized geometries of **1b** and **1c** with the proper substitutions. SHG hyperpolarizabilities, that is, the *β*(–2*ω*;*ω*,*ω*) tensors, were computed in CHCl_3_ within the coupled perturbed Kohn–Sham (CPKS) approach, at the same frequency as used as in the Kurtz–Perry experiments. The CAM-B3LYP functional [72], which has been recommended for hyperpolarizability calculations of mid-size organic chromophores [73], was adopted. The solvent was described as a continuum dielectric according to the polarizable continuum model in its integral equation formalism variant (IEFPCM algorithm) [74]. A pruned (99,590) grid was selected for computation as well as the use of two-electron integrals and their derivatives. As already pointed out in our previous studies on the NLO properties of [Cu(5-A-5′-D-salen/saltn)] [38] and [Ni(5-A-5′-D-saltn)] [39] complexes, two main and almost orthogonal components of the dipole moment and hyperpolarizability vectors can be singled out in these compounds, one along the largest molecular extension and the other across the ON(py)/O_2_ and N_2_ atoms of the donor set of compounds **1** and **2**. In order to meaningfully compare these components among the examined compounds, a common reference frame was adopted, whereby the origin coincides with the copper ion, the *x* axis passes through C4 (the carbon atom of the six-membered chelate ring on the acceptor side) and points from the acceptor towards the donor group, the *y* axis lies in the N_3_O/N_2_O_2_ plane and points from the ON(py)/O_2_ towards the N_2_ atoms of the donor set, and the *z* axis completes the right-handed reference system. This frame strictly recalls that adopted for the [Cu(5-A-5′-D-salen/saltn)] [38] and [Ni(5-A-5′-D-saltn)] [39] complexes.

### 2.10. Kurtz–Perry Powder Measurements

The measurements of SHG intensity were carried out by the Kurtz–Perry powder technique [47] using a nanosecond Nd:YAG pulsed (10 Hz) laser. The fundamental beam (1.064 μm) was focused in a hydrogen cell (1 m long, 50 atm) and the outcoming Stokes-shifted radiation generation at 1.907 μm was used as the fundamental beam for SHG. The SHG signal coming from capillary tubes containing grinded microcrystalline powders (50–80 μm) of the samples was detected by a photomultiplier.

## 3. Results and Discussion

### 3.1. Synthesis and IR/MS Characterization

Even if it is reported that tridentate HL ligands, especially with chxn, can be obtained without the support of a metal ion [56,75], because of our interest in copper(II) derivatives, we decided to directly employ the template method that was revealed to be efficient with aliphatic diamines en and tn [38,76,77,78], as well as with the aliphatic diamines chxn and dpen. The series of mononuclear derivatives **1a**–**c** with tridentate Schiff base ligands was then synthesized by reaction of NO_2_-salH:Cu(ClO_4_)_2_:diamine in a 1:1:1 ratio in the presence of excess py acting as the forth ligand, so as to avoid the double condensation on both NH_2_ groups of chxn and dpen. The cheapest (1*S*,2*R*)-*meso*-dpen was initially employed with the aim of studying the best reaction and isolation conditions, and then extending the synthetic protocol to the most expensive optically pure (1*R*,2*R*)-(+)-dpen and (1*S*,2*S*)-(+)-chxn. It is curious to note how we could instead observe a moderate different reactivity of (1*S*,2*R*)-*meso*-dpen compared with (1*R*,2*R*)-(+)-dpen. In fact, the species [Cu(L^b^)(H_2_O)](ClO_4_), **1b’**, could be isolated with (1*R*,2*R*)-(+)-dpen, where the crystal packing of cations and anions in the solid state seems to confer a lower solubility to the aquo complex with its preferential precipitation. A similar complex with NO_2_-salH, en and water as the forth coordinated ligand was also isolated by us in our previous work [77]. Only in the presence of a larger excess of pyridine, **1b’** was converted into **1b** with coordinated py. Intermediates **1b** and **1c** were then suitably converted to **2a**, **2b**, and **2c** by condensation with the second carbonyl derivative, salH or 5-OMe-salH, in refluxing MeOH; the latter aldehyde, owing to the donor group, is characterized by a higher reactivity with a consequent lower reaction time (30 min) compared with salH (1 h).

All compounds were characterized by infrared spectroscopy, where the N–H stretching bands in the 3350–3250 cm^−1^ spectral range are diagnostic for the formation of intermediates **1a**–**c** and **1b’**. The disappearance of such bands in the condensation reaction to obtain **2a**–**c** could be used as a probe of the complete conversion of the intermediates to the final tetradentate derivatives. The C=N stretching bands are also subjected to peculiar changes on going from 1645–1647 cm^−1^ in **1b** and **1c** to 1632–1635 in **2a**–**c**. In all derivatives, the stretching band of the nitro group at about 1315 cm^−1^ is detectable. The coordinated water molecule in **1b’** can be recognized through the narrow O–H stretching band at 3296 cm^−1^ in between the symmetric and asymmetric NH_2_ bands. Furthermore, the different donating power to copper(II) of the oxygen atom in the water molecule compared with the nitrogen atom of py leads to modifications in the stretching bands of the nitro group, which shifts to 1333 cm^−1^, and of the C=N bond, which moves to 1626 cm^−1^.

The chemical purity of bulk materials was proved by elemental analyses and all compounds were also subjected to MS-ESI investigations. In particular, mass peaks at *m/z* corresponding to the [Cu(L^a–c^)]^+^ and solvated [Cu(L^a–c^)(MeOH)]^+^ fragments could be detected for **1a**, **1b**, and **1c**, while the most intense peak of **1b’** was the [Cu(L^b^)(H_2_O)]^+^ ion, suggesting a rather strong Cu–O(water) bond. In the case of **2a**, **2b**, and **2c**, mass peaks at *m/z* corresponding to [M + 1]^+^ and [M + Na]^+^ species, together with the dimeric [2M + 1]^+^ and [2M + Na]^+^ ions, were observed. It is not uncommon to observe such dimeric signals in tetradentate Schiff base metal complexes owing to the establishment of short intermolecular M∙∙∙O(phenol) interactions [33], especially with copper(II) and its tendency to weakly interact with a fifth donor atom [38,77].

### 3.2. X-ray Structures of **1a**∙0.5H_2_O, **1b**, and **1c**

**Figure 1:**
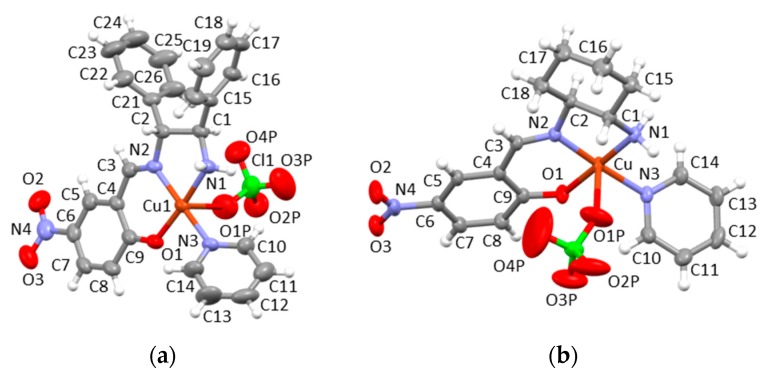
X-ray structures with atom numbering scheme of (**a**) one of the two independent cations of **1b** with one interacting perchlorate anion and (**b**) **1c**. Displacement ellipsoids are drawn at 30% probability; color code: Cu = orange, Cl = green, O = red, N = light blue, C = grey, H = white.

Single crystals suitable for XRD experiments were obtained for **1a**∙0.5H_2_O, **1b**, and **1c** bearing the tridentate Schiff base ligands L^−^. The molecular structures of **1b** and **1c** are reported in Figure 1, while a fragment of the crystal packing of **1a** is depicted in Figure S3 in SM with an atom numbering scheme. Selected bond distances, angles, and other structural parameters of all compounds helpful for the discussion are reported in Table 2. Compound **1a** crystallizes in the centrosymmetric *P*2_1_/*n* space group, because (1*S*,2*R*)-*meso*-dpen can condense the amino group with NO_2_-salH on either the *R* or *S* chiral side, and this gives rise to two enantiomeric complexes, both present in the unit cell and related by the inversion center. When moving to (1*R*,2*R*)-(+)-dpen and (1*S*,2*S*)-(+)-chxn, only one pure enantiomer is present, so that **1b** and **1c** crystallize in the orthorhombic *P*2_1_2_1_2_1_ and the chiral tetragonal *P*4_3_ space groups, respectively.

All complexes show the expected square-planar geometry around the copper(II) atoms, where the basal plane is formed by the N_2_O set of donors from the L^a−^, L^b−^, and L^c−^ ligands for **1a**, **1b**, and **1c**, respectively, and the fourth position is occupied by the nitrogen atom of the pyridine ligand. All complexes are characterized by very slight tetrahedral distortion of the donor set, as indicated by the values of the O1–N2–N1–N3 torsion angles, all less than 1.0°, except for the second molecule of the asymmetric unit of **1b**, where the O4–N6–N5–N4 torsion angle measures 7.9°. The displacement of the copper(II) ions from the N_3_O l.s. planes is very low in all cases (<0.07 Å). Coordination distances and angles are comparable to those of other copper compounds with N_2_O tridentate Schiff base ligands and pyridine as the forth ancillary ligand [77,79].

The asymmetric units of **1a** and **1c** comprise one independent [Cu(L^a/c^)(py)]^+^ cation and ClO_4_^−^ anion, together with a co-crystallized half molecule of water in the case of **1a**. Analysis of the crystal structure of **1a** reveals that the perchlorate anion is disordered over three positions: a majority one (labelled with R, Figure S3a in SM), which is placed far from the metal and is hydrogen-bonded to one iminic hydrogen atom (N1–H1B∙∙∙O3R, r_H∙∙∙O_ = 2.29 Å, NH∙∙∙O angle = 138.1°) and a CH group of the sal moiety (C3–H3∙∙∙O2R, r_H∙∙∙O_ = 2.59 Å, CH∙∙∙O angle = 156.5°); and a minority one, which is in turn disordered over two almost overlapped positions (labelled as P and Q) with equal occupancy, one of them weakly interacting with copper (Cu1∙∙∙O1P = 2.586 Å, Figure S3b in SM). The water molecule is complementary to the perchlorate anion: when the most populated ClO_4_^−^ site is occupied, the oxygen atom is placed at Cu1∙∙∙O1W = 2.57(1) Å from copper and is hydrogen-bonded, on the other side, with ClO_4_^−^ (r_O1W∙∙∙O1R_ = 2.854 Å); otherwise, it is hydrogen-bonded to the iminic hydrogen atom (N1–H1B∙∙∙O2W, r_H∙∙∙O_ = 2.18 Å, NH∙∙∙O angle = 152.6°).

**Figure 2:**
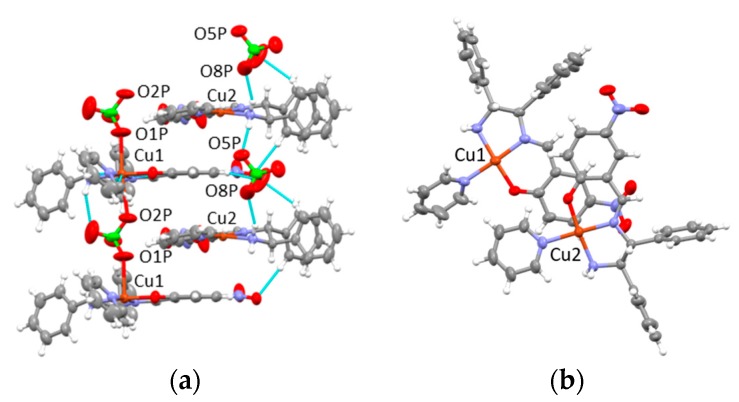
Fragments of crystal packing in **1b** viewed along (**a**) *c* and (**b**) *a*; displacement ellipsoids are drawn at 30% probability; color code: Cu = orange, Cl = green, O = red, N = light blue, C = grey, H = white (intermolecular contacts are shown as cyan lines; perchlorate anions are not shown in (**b**) for clarity).

The asymmetric unit of **1b** contains two independent [Cu(L^b^)(py)](ClO_4_) ionic pairs. In one of them (Cu1), the pyridine ring is twisted with respect to the coordination plane (54.2(3)°), as also observed in **1a** (63.0(1)°) and **1c** (58.5(1)°). Short intermolecular contacts are observed between the copper ion and perchlorate anions on both sides of the coordination plane (Cu1∙∙∙O1p = 2.418(5) and Cu1∙∙∙O2p’ = 2.557(5) Å), forming linear 1D chains ∙∙∙Cu1∙∙∙O1p–Cl–O2p∙∙∙Cu1′∙∙∙ along the crystallographic *a* axis (chain A). The second independent cation (Cu2) shows a lower rotation of the pyridine with respect to the coordination plane (14.1(1)°), in absence of short Cu∙∙∙OClO_3_ contacts, while the perchlorate anion is involved in rather strong hydrogen bonds with the coordinated amino group (N5–H5B∙∙∙O5p, *r*_H∙∙∙O_ = 2.15 Å, NH∙∙∙O angle = 172.1°; N5–H5A∙∙∙O8p, *r*_H∙∙∙O_ = 2.49 Å, NH∙∙∙O angle = 158.6°), yielding a second supramolecular 1D alignment (chain B). The two chains are then inserted one in the other with weak C–H∙∙∙O hydrogen bonds connecting aromatic units of one chain with either the nitro moiety or the perchlorate anion of the adjacent chain (Figure 2a). The angle between coordination l.s. planes of Cu1 and Cu2 is equal to 9.26°. Most importantly, in view of the analysis of the NLO response, cations are iso-oriented along each chain, according to a head-to-head arrangement, while stacked cations between A and B chains are rotated by about 90° with respect to the each other (see Figure 2b).

**Figure 3:**
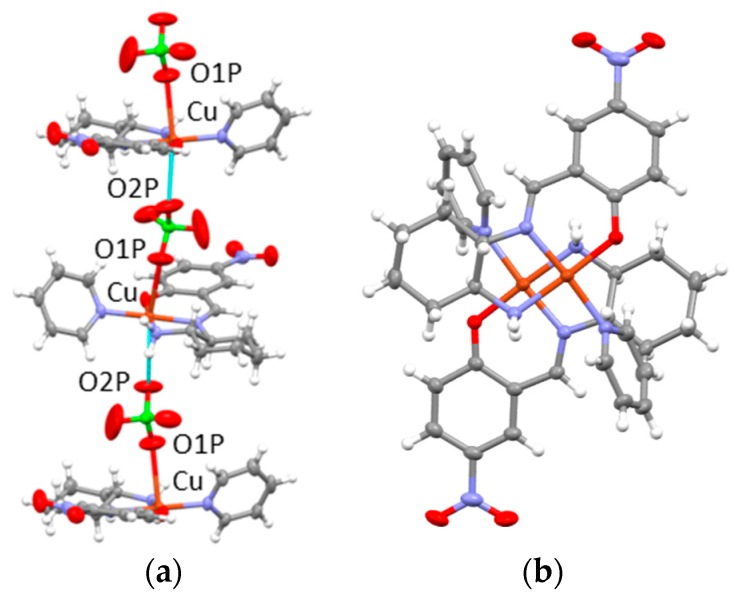
Fragments of crystal packing in **1c** viewed along (**a**) *a* and (**b**) *c*; displacement ellipsoids are drawn at 30% probability; color code: Cu = orange, Cl = green, O = red, N = light blue, C = grey, H = white (intermolecular contacts are shown as cyan lines; perchlorate anions are not shown in (**b**) for clarity).

Short contacts with the perchlorate anions on both sides of the coordination plane can also be detected in **1c** (Cu1∙∙∙O1p = 2.654(5) Å, Cu1∙∙∙O2p = 2.857(5) Å), even if slightly longer than in **1b**, thus producing a zig-zag 1D chain along the crystallographic *c* axis with an angle between three consecutive copper(II) ions of 159.1° (Figure 3a). Along this direction, cations are almost parallel (the angle between the coordination l.s. planes of two consecutive cations measures 16.94°) and oriented in the opposite direction (see Figure 3b), suggesting a detrimental effect on the NLO response in the crystal phase. The amino group in **1c** is involved in a short hydrogen bond with the nitro group of an adjacent molecule (N1–H1A∙∙∙O3′, r_H∙∙∙O’_ = 2.28 Å, NH∙∙∙O’ angle = 157.9°), which is further engaged in short contacts with the pyridine ring (O3′∙∙∙C13 = 3.203(5) Å, O3′∙∙∙C14 = 3.155(4) Å), giving rise to the inter-locked mode between parallel chains. This arrangement forms 2D layers parallel to the *ab* plane, where the molecules of each layer are alternately oriented in the same direction.

### 3.3. Absorption Spectroscopy

**Figure 4:**
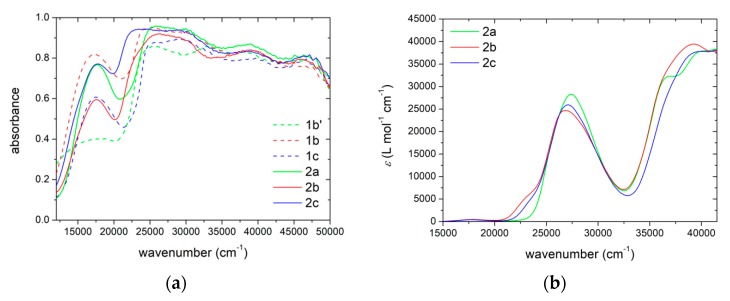
UV/visible absorption spectra of (**a**) **1b**, **1b’**, **1c**, **2a**, **2b**, and **2c** in the solid state and (**b**) **2a**, **2b**, and **2c** in 2 × 10^−5^ CHCl_3_ solutions (zoom of the *d*–*d* transition region from 12,500 to 20,000 cm^−1^ can be found in Figure S4 in SM).

The electronic spectra in the solid state of **1b**, **1b’**, **1c**, **2a**, **2b**, and **2c** are reported in Figure 4a in the 12,000–50,000 cm^−1^ (900–200 nm) region. In all cases, it is possible to recognise a well-isolated *d*–*d* transition in the 17,300–17,800 cm^−1^ (562–578 nm) range, with the exception of **1b’**, in which the band appears to be much broader and weaker. This clearly reflects the different ligand field given by the substitution of a water molecule by a pyridine ligand. Charge transfer (CT) and π → π* transitions start to appear above 20,000 cm^−1^. The introduction of the OMe group on going from **2a** to **2b** leads to the absorption at lower wavenumbers, as expected for the favoured ligand-to-metal charge transfer (LMCT) transition by the presence of the donor group [38,39]. When moving to **2c**, even if the push-pull substitution is equal to **2b**, the LMCT band moves further to lower energies, which might be caused by the presence of close molecules with short intermolecular interactions [53], favoured by the smaller steric hindrance of the cyclohexyl ring compared with the two phenyl rings on the diamine bridge [60]. The same behaviour is present when comparing **1b** and **1c**, where the donor sal moiety is absent, which can thus confirm the occurrence of favoured electronic transitions in chxn derivatives. Above 30,000 cm^−1^, all spectra appear very similar owing to further π → π* transitions.

Solution studies were performed on non-ionic derivatives **2** in CHCl_3_ solutions from 10^−3^ to 2 × 10^−5^ mol L^−1^ (see Figure 4b). The *d*–*d* transition in **2a**, **2b**, and **2c** appears as a relatively weak band at about 17,730–17,860 cm^−1^ (560–564 nm) with *ε* ~ 500 L mol^−1^ cm^−1^ (see Figure S4 in SM for a zoom of the 12,500–20,000 cm^−1^ region), as expected for copper(II) complexes with a rigid square planar geometry given by both chiral [51,52,54] and achiral [38] N_2_O_2_ salen-type ligands, with no apparent effect given by the diamine bridge or substituents. Position, shape, and intensity of the *d*–*d* band also follow the Lambert–Beer law, which suggests the absence of stacked molecules in solution at a concentration lower than 10^−3^ mol L^−1^. In the 20,000–32,000 cm^−1^ range, there is an intense absorption with *ε* coefficients as high as 27,000 L mol^−1^ cm^−1^, which can be attributed to a convolution of CT transitions [52,80]. In particular, the position of the LMCT band depends on the substituents present on the molecular skeleton, as confirmed by its visibility in the lowest energy side as a shoulder at about 22,500 cm^−1^ (*ε* ~ 4000 L mol^−1^ cm^−1^) in **2b** and **2c** bearing the OMe group, while it moves below the strongest intra-ligand charge transfer (ILCT) π → π* transitions in **2a**, where there is no donor substituent. In fact, we previously confirmed the nature of the LMCT band on derivatives with non-chiral en and tn bridges, where the HOMO orbital depicted by theoretical calculations is mainly localized on the D-sal moiety [38,39]. In the UV region above 33,000 cm^−1^, a strong absorption due to further π → π* transitions appears [80], with noticeable differences among the three complexes. In particular, compounds with dpen possess a stronger absorbance at lower energies, probably given by transitions where the aromatic rings of the diamine bridge are involved. Comparing **2a** and **2b**, the latter shows a more pronounced maximum at 39,370 cm^−1^ (254 nm) and *ε* = 40,600 L mol^−1^ cm^−1^, promoted by the presence of the OMe group.

Ionic complexes **1** are not soluble in non-coordinating solvents, while they are, for example, in methanol. Nevertheless, they probably undergo substitution of the pyridine ligand once in MeOH [78,81], so that the spectrum corresponds to the solvated species [Cu(L)(MeOH)]^+^. To overcome this drawback, studies in solution were performed in MeOH (from 10^−3^ to 4 × 10^−5^ mol L^−1^), adding increasing amounts of pyridine to the most diluted solution. The obtained spectra of **1b** and **1c** in MeOH (reported in Figure S5 in SM) are not too dissimilar from those of derivatives **2**, except for shifted (16,050 cm^−1^, 623 nm) and weaker (135 L mol^−1^ cm^−1^) *d–d* transitions. Upon addition of pyridine, this transition moves toward higher energies, 17,670 cm^−1^ (566 nm), and higher intensity, *ε* = 300 and 235 L mol^−1^ cm^−1^, for **1b** and **1c**, respectively. This seems to confirm the [Cu(L)(MeOH)]^+^ → [Cu(L)(py)]^+^ back conversion in solution, owing to the stronger ligand field given by pyridine with respect to MeOH. Nevertheless, the LMCT + ILCT transitions are not affected by the coordination of either py or MeOH.

### 3.4. DFT Structural, Electronic, and NLO Properties

In view of theoretically evaluating the molecular nonlinear optical properties of the investigated compounds, DFT geometry optimizations were performed on the cations of **1a**–**c**. The experimental bond lengths were satisfactorily reproduced (see Table 2), with the exceptions of only the Cu–N1 and Cu–N3 distances, which are systematically longer in the optimized structures. This reflects the greater tendency of such bonds to be deformed by packing forces with respect to the other coordinating bonds. For the same reason, the dihedral angles between the N_3_O and pyridine l.s. planes, which are virtually the same in the optimized structures, are very different from the X-ray values, displaying large variations not only from one structure to another, but also in the same structure, as observed in **1b**. The comparison of the coordinating bond lengths in **1a**–**c** does not reveal significant differences, as expected from the analogous electronic environment around the copper ion in the three structures.

**Figure 5:**
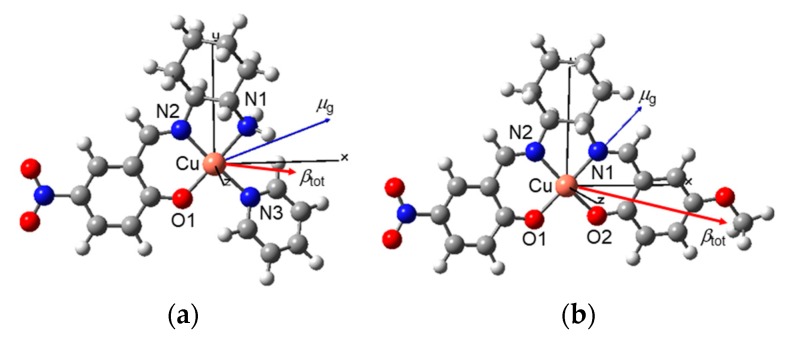
Optimized geometries of (**a**) cation of **1c** and (**b**) **2c** with *μ*_g_ and *β*_tot_ vectors expressed in arbitrary units; color code: Cu = light orange, O = red, N = blue, C = grey, H = white.

DFT geometry optimizations were also performed on **2a**–**c**, for which X-ray data were not available, owing to the well-assessed reliability of DFT in reproducing the molecular features of this class of complexes [38,39]. As we previously observed and theoretically reproduced in the [Cu(5-A-5′-D-salen)] derivatives [38], the NO_2_ group is more efficient in modulating the coordination geometry with respect to the OMe one. Such influence is mainly manifested in the Cu–O bonds, with elongation of Cu–O1 (A side) and a concomitant decrease of Cu–O2 (D side) bond lengths. The Cu–N bonds are less affected by the presence of the NO_2_ group, with a slightly more elongated Cu–N2 (A side) with respect to Cu–N1 (D side) bond lengths (Table 3, Figure 5, and Figure S6 in SM). Comparing **2b** and **2c**, having the same (NO_2_, OMe) push-pull pattern, but a different diamine (dpen vs. chxn, respectively) bridge, the most remarkable difference lies in the dihedral angle between the l.s. planes through the two sal moieties, which is higher in **2c** (12.33°) with respect to **2a** (10.20°) and **2b** (10.28°). The reason for this difference can be probably found in the greater steric demand of the cyclohexyl ring with respect to the dpen bridge.

As expected, the cations of compounds **1a**, **1b**, and **1c** are very similar from an electronic point of view (see Table 4). Using the copper ion as the origin of the Cartesian reference (see computational details), their dipole moments, about 13.5 D, and hyperpolarizability vectors *β*_tot_, about 23–24 × 10^−30^ cm^5^ esu^−1^, reflect an almost indistinguishable behavior of the compounds at the molecular level, in both the ground and the excited state. Compounds **2a**, **2b**, and **2c**, on the other hand, while having similar dipole moments (13.02, 12.26, and 12.34 D), show increasing values of *β*_tot_ from 39.7 to 49.2 and 49.6 × 10^−30^ cm^5^ esu^−1^, going from **2a** to **2b** and **2c**, as a consequence of the presence of the donor group OMe in the latter compounds. These values are quite similar to those previously obtained for the [Cu(5-A-5′-D-salen)] analogues with (NO_2_, H) and (NO_2_, OMe) substitutions [38,39], indicating, at the molecular level, the almost irrelevant influence of the ethylene substituents (en vs. dpen vs. chxn) on the electronic properties.

### 3.5. Solid-State NLO Properties

The solid-state NLO response of powder samples **1b**, **1c**, **2a**, **2b**, and **2c** was investigated using the Kurtz–Perry technique [47] @1.907 µm, resulting in SHG efficiencies equal to 0.13, 0.44, 0.32, 0.19, and 0.30 times that of standard urea, respectively. Even if not high, these values are in line with those reported for the majority of similar Schiff base metal complexes [45,46,64,65,84], and lower than those reported only for [Mn(Cl)(4,4′-diNEt_2_-sal-(1*R*,2*R*)-chxn)] [23,48] and [Ni(4,4′-diNEt_2_-sal-(1*R*,2*R*)-dpen)] [23,49], whose SHG efficiencies are up to 8 and 13 times that of urea, respectively.

Owing to the absence of the crystal structure of **2a**–**c**, only hypothesis to compare the molecular properties as described by theoretical calculations with the solid-state NLO data can be formulated in the case of derivatives with unsymmetrically-substituted tetradentate ligands. A possible explanation for the observed diminished *χ*^(2)^ is the unfavorable alignment of the chromophores, which probably still tend to couple in a head-to-tail fashion, in agreement with previous reports [38,39]. Anyway, the chirality conferred to the final complexes by the diamines prevents perfect alignment, resulting in a small, but non-zero solid-state NLO response. In particular, theoretical calculations gave reasonably similar *β*_tot_ values (49.22 and 49.60 × 10^−30^ cm^5^ esu^−1^) for **2b** and **2c**, respectively, bearing the same A–D substitution, while their SHG efficiencies are 0.19 and 0.30 times that of standard urea, which suggests a better performance of the chxn diamine bridge with respect to dpen. This is in opposition to what was observed in the case of [Ni(4,4′-diNEt_2_-sal-(1*R*,2*R*)-chxn)] [48] and [Ni(4,4′-diNEt_2_-sal-(1*R*,2*R*)-dpen)] [49], where a higher SHG efficiency was achieved by the latter compound (9 vs. 0.3 times that of standard urea for 50–80 μm grain caliber) and ascribed to its higher degree of chirality [85] conferred by the spatial disposition of the two phenyl rings [49]. The opposite chirality of chxn employed in **2c** with respect to [Ni(4,4′-diNEt_2_-sal-(1*R*,2*R*)-chxn)] does not have an effect because the solid-state NLO response depends on the molecular hyperpolarizability, the orientation of the molecule with respect to the crystalline reference frame, and the nature of the crystal point group [10]—properties that are coincident with those of the enantiomer of **2c**, that is, [Cu(5-NO_2_-5′-OMe-sal-(1*R*,2*R*)-chxn)].

The same trend in the SHG efficiency can be drawn out for **1b** (dpen) and **1c** (chxn), where their experimental values are 0.13 and 0.44 times that of standard urea against similar computed *β*_tot_ values of 22.98 and 24.08 × 10^−30^ cm^5^ esu^−1^, respectively. These values can be interpreted through both the analysis of degree of chirality and the relationship between microscopic *β_xyz_* and macroscopic *β_XYZ_*, with both pieces of information being affordable for these compounds thanks to their known crystal structure. Quantification of the degree of chirality with the continuous symmetry measures (CSM) formalism proposed by Avnir et al. [86], on a scale from 0 (achiral) to 100, gave a greater value in **1c** (7.7948, only the cation: 1.9812) than in **1b** (chain A: 4.3929, cation: 1.5796; chain B: 6.4284, cation: 0.5959), considering either the cations [Cu(L)(py)]^+^ or the [Cu(L)(py)](ClO_4_) assemblies. This would suggest a kind of correlation between solid-state NLO response and the degree of chirality.

On the other side, the relationships between microscopic and macroscopic optical nonlinearities can be derived considering that crystals of **1b** belong to the *P*2_1_2_1_2_1_ orthorhombic space group with two cations in the asymmetric unit. For the 222 point group, there is only one phase matchable coefficient, *β_XYZ_* = sin2*Φ* cos*θ* (*β_xyy_* − *β_xxx_* sin^2^*θ*) − *β_yxx_* cos2*Φ* sin2*θ* [10], where *Φ* is the projection of the CT axis on the *ab* plane and *a*, and *θ* is the angle between the CT axis and *c* [87]. The optimal value of the angular factor is 0.192, reached for *Φ* = 45° and *θ* = 54.7°. On the basis of CP-DFT calculations on the **1b** cation, the CT axis lies approximately along the C4–Cu direction (i.e., the *x* axis, see Figure S6b in SM and Table 4), so that *Φ* = 89° and *θ* = 75° for molecule 1 of chain A in the asymmetric unit, while *Φ* = 84° and *θ* = 32° for molecule 2 of chain B. As only *β_xxx_* is significantly different from zero for **1b** cation, both values of *Φ*, close to 90°, result in very small macroscopic susceptibility *β_XYZ_*. In the case of **1c** (tetragonal space group *P*4_3_, point group 4), the only non-zero macroscopic nonlinear susceptibilities are *β_ZZZ_* = *β_xxx_*cos^3^*θ* and *β_ZYY_* = (*β_xyy_* +*β_xxx_* sin^2^*θ*) cos*θ*/2, *θ* being the angle between the CT axis and the four-fold axis *Z*. The almost orthogonality between these two axis (*θ* = 82°), far from the optimal value *θ* = 54.7°, explains the low NLO susceptibility of **1c**.

## 4. Conclusions

The synthesis, structural and spectroscopic characterizations, and SHG efficiencies using the Kurtz–Perry technique of mononuclear copper(II) complexes with either tridentate or tetradentate Schiff base ligands are reported here with the aim of investigating their NLO performances to the solid state. The synthetic copper(II)-templated approach for tridentate Schiff base ligands L^−^ derived from salicylaldehydes and aliphatic diamines [77] is efficiently exploited here with the use of the chiral diamines chxn and dpen, allowing also to isolate the intermediate **1b’** with coordinated water molecule, further replaced by pyridine. Derivatives [Cu(L^a–c^)(py)](ClO_4_) (**1a**–**c**) were then converted into compounds [Cu(5-A-5′-D-saldpen/chxn)] (**2a**–**c**) with chiral tetradentate Schiff base ligands, obtaining push-pull complexes in which the metal ion promotes an intense LMCT transition, as observed in the UV/vis absorption spectroscopy.

Despite the low solid-state NLO efficiencies of about 0.15–0.45 times that of standard urea for all derivatives, these values are in line with other similar compounds, with the exclusion of only a few exceptional cases [48,49]. X-ray structure determination of **1b** and **1c**, together with theoretical calculations on their NLO molecular response, allowed to ascribe their modest NLO susceptibilities to the non-optimal alignment of their CT axes with respect to the crystalline reference frame.

## Figures and Tables

**Table 1:** Crystallographic data for **1a**∙0.5H_2_O, **1b**, and **1c**.

	1a∙0.5H_2_O	1b	1c
*Crystal Data*			
Moiety formula	[C_26_H_23_CuN_4_O_3_](ClO_4_)∙0.5H_2_O	[C_26_H_23_CuN_4_O_3_](ClO_4_)	[C_18_H_21_CuN_4_O_3_](ClO_4_)
Sum formula	C_26_H_24_ClCuN_4_O_7.5_	C_26_H_23_ClCuN_4_O_7_	C_18_H_21_ClCuN_4_O_7_
*M*	611.47	602.47	504.38
Crystal system	monoclinic	orthorhombic	tetragonal
Space group	*P*2_1_/*n* (n. 14)	*P*2_1_2_1_2_1_ (n. 19)	*P*4_3_ (n. 78)
*a*/Å	13.232(3)	7.1810(7)	11.6613(6)
*b*/Å	13.329(3)	21.970(2)	11.6613(6)
*c*/Å	15.383(3)	35.121(3)	15.3217(8)
*α*/°	90	90	90
*β*/°	92.40(3)	90	90
*γ*/°	90	90	90
*V*/Å^3^, *Z*	293(2), 4	5541.0(9), 8	2083.5(2), 4
Reflns for cell det	130	6497	9386
*2θ/*° for cell det	16.0–52.0	4.64–40.24	4.39–60.26
*D_x_*/Mg m^−3^	1.515	1.444	1.608
*μ*/mm^−1^	1.318	0.935	1.226
Colour, habit	green, needle	violet, needle	red, block
Dimensions/mm	0.20 × 0.01 × 0.01	0.58 × 0.14 × 0.08	0.36 × 0.34 × 0.30
*Data Collection*			
Temperature/K	293(2)	293(2)	294(2)
radiation *λ*/Å	synchrotron, 0.800	Mo–K*α*, 0.71073	Mo–K*α*, 0.71073
Scan type	*φ*	*φ* and *ω*	*φ* and *ω*
2*θ*_max_/°	57.4	49.7	64.5
*h* range	−16 → 16	−8 → 8	−17 → 17
*k* range	−17 → 17	−25 → 25	−17 → 17
*l* range	−17 → 17	−41 → 41	−22 → 22
Intensity decay	None	None	None
Measured reflns	31,811	66,969	44,359
Independent reflns	4628	9600	7153
Reflns with *I* > 2*σ*(*I*)	4001	7633	6226
*R*_int_	0.0390	0.0415	0.0214
*Refinement on F* ^2^			
*R_1_*, *wR_2_* [*F*^2^ > 2*σ*(*F*^2^)]	0.0429,0.1165	0.0430, 0.1073	0.0321, 0.0901
*R_1_*, *wR*_2_ [all data]	0.0499, 0.1231	0.0604, 0.1176	0.0394, 0.0949
*S*	1.101	1.016	1.029
Flack parameter		0.007(5)	0.014(3)
Params, restraints	455, 159	703, 0	280, 1
(Δ*/σ*)_max_	0.001	0.001	0.001
Δ*ρ*_max_, Δ*ρ*_min_/e Å^−3^	0.293, −0.343	0.375, −0.288	0.396, −0.345

**Table 2:** Selected interatomic distances (Å) and angles (°), intermolecular contacts (Å), and selected parameters in **1a**∙0.5H_2_O, **1b**, and **1c** from single crystal XRD (first line) and UM06/6-311++G(d,p) calculations on cations (second line, in italics; see Figure 1 and Figure S3 in SM for atom labelling).

	1a∙0.5H_2_O	1b	1c
Cu1–N1/Cu2–N5 ^1^	2.011(5)	1.998(4)/2.002(5) ^1^	2.007(2)
	*2.066*	*2.054*	*2.060*
Cu1–N2/Cu2–N6 ^1^	1.949(4)	1.958(4)/1.951(4) ^1^	1.951(2)
	*1.940*	*1.948*	*1.952*
Cu1–O1/Cu2–O4 ^1^	1.903(4)	1.901(4)/1.873(4) ^1^	1.902(2)
	*1.899*	*1.896*	*1.894*
Cu1–N3/Cu2–N7 ^1^	1.994(5)	2.011(5)/2.032(5) ^1^	2.015(2)
	*2.014*	*2.030*	*2.028*
Cu1∙∙∙O1p, Cu1∙∙∙O2p ^2^	2.59(1), absent	2.418(5), 2.557(5) ^2^	2.654(5), 2.857(5)
N1–Cu1–N2/N5–Cu2–N6 ^1^	83.38(18)	83.43(18)/83.27(18) ^1^	84.36(9)
	*83.44*	*83.35*	*83.49*
N2–Cu1–O1/N6–Cu2–O4 ^1^	92.65(17)	93.60(18)/92.27(18) ^1^	93.58(9)
	*93.31*	*93.52*	*93.84*
O1–Cu1–N3/O4–Cu2–N7 ^1^	87.86(18)	89.25(18)/88.9(2) ^1^	91.02(9)
	*89.84*	*88.45*	*88.76*
N3–Cu1–N1/N7–Cu2–N5 ^1^	96.19(19)	93.46(19)/96.2(2) ^1^	91.02(10)
	*95.29*	*94.69*	*94.44*
N1–Cu1–O1/N5–Cu2–O4 ^1^	173.86(18)	175.0(2)/172.8(2) ^1^	177.24(10)
	*168.50*	*175.78*	*171.61*
N2–Cu1–N3/N6–Cu2–N7 ^1^	178.8(2)	175.3(2)/174.0(2) ^1^	175.36(9)
	*169.69*	*178.02*	*175.51*
Cu1∙∙∙N_3_O/Cu2∙∙∙N_3_O (Å) ^1^	0.030	0.066/0.006 ^1^	0.020
	*0.005*	*0.022*	*0.034*
N_3_O ∠ py (°)	63.0(1)	54.2(3)/14.1(1) ^1^	58.5(1)
	*41.27*	*36.54*	*38.86*

^1^ the second set of atom names and parameters are referred to the second independent molecule of **1b**; ^2^ the Cu2 atom in **1b** is not involved in short intermolecular contacts with perchlorate anions.

**Table 3:** Selected interatomic distances (Å) and angles (°) in **2a**, **2b**, and **2c** from UM06/6-311++G(d,p) calculations (see Figure 5 and Figure S6 in SM for main atom labelling).

	2a (NO_2_, H)	2b (NO_2_, OMe)	2c (NO_2_, OMe)
Cu–N1 ^1^	1.952	1.954	1.957
Cu–N2 ^1^	1.966	1.967	1.969
Cu–O1 ^1^	1.928	1.913	1.931
Cu–O2 ^1^	1.903	1.897	1.895
O1–Cu–O2	90.51	90.62	91.23
N1–Cu–N2	84.34	84.30	84.02
A–sal ∠ D–sal ^2^	10.20	10.28	12.33

^1^ O1 and N2 on the acceptor side, O2 and N1 on the donor side; ^2^ dihedral angle between the l.s. plane through the nine C/N/O atoms of the two sal moieties, excluding A or D.

**Table 4:** Computed^1^ dipole moments (*μ*_g_, *μ*_x_, *μ*_y_, and *μ*_z_, D), SHG (*λ* = 1.907 μm) hyperpolarizabilities (*β*_tot_, *β*_vec_, *β*_x_, *β*_y_ and *β*_z_, 10^−30^ cm^5^ esu^−1^) in CHCl_3_, and angle (*θ*, °) between *μ*_g_ and *β*_tot_ vectors for cations of **1a**–**c** and for **2a**–**c**.

	1a	1b	1c	2a	2b	2c
*μ*_x_	12.66	12.34	12.66	7.86	8.47	8.48
*μ*_y_	4.69	5.42	4.71	10.35	8.81	8.96
*μ*_z_	−2.12	−0.56	0.71	0.88	0.95	0.12
*μ*_g_	13.66	13.49	13.53	13.02	12.26	12.34
*β*_x_	23.72	22.79	23.61	37.70	47.02	48.75
*β*_y_	−4.11	−2.78	−3.01	−12.44	−14.50	−8.98
*β*_z_	0.64	−1.06	0.56	−0.65	−0.77	−1.93
*β*_tot_	24.08	22.98	23.81	39.71	49.22	49.60
*β*_vec_	20.46	19.78	21.08	12.83	22.00	26.98
*θ*	31.8	30.6	27.7	71.2	63.5	57.0

^1^ Calculations were performed at the (CP)CAM-B3LYP/6-311++G(d,p)/IEFPCM(CHCl_3_) level of theory on the UM06/6-311++G(d,p) optimized geometries; see computational details section for the adopted reference frame to report the *μ*_i_ and *β*_i_ components; hyperpolarizabilities are defined as *β*_tot_ = (*β*_x_^2^ + *β*_y_^2^ + *β*_z_^2^)^1/2^ and *β*_vec_ = Σ_i_ (*μ*_i_ *β*_i_)/|*μ*|, where *β*_i_ = (1/3) Σ_j_ (*β*_ijj_ + *β*_jij_ + *β*_jji_) [82]. In particular, *β*_tot_ = (5/3) *β*_‖_ and *β*_vec_ = (5/3) *β*_‖_(z), where *β*_‖_ and *β*_‖_(*z*) are the quantities printed in the Gaussian16 output, according to the Taylor convention [83].

## Supplementary Materials

The following are available online at https://www.mdpi.com/1996-1944/12/21/3595/s1, Figure S1: MS-ESI+ spectra of compounds **1a**–**c**, **1b’** and **2a**–**c** registered in MeOH solution; Figure S2: FT-IR spectra of compounds **1a**–**c**, **1b’**, and **2a**–**c** registered as KBr disks; Figure S3: X-ray crystal structure of **1a**∙0.5H_2_O with atom numbering scheme; Figure S4: UV/visible absorption spectra in the 12,500–20,000 cm^−1^ range (*d*-*d* transition region) of **2a**, **2b**, and **2c** in 10^−3^ mol L^−1^ CHCl_3_ solutions; Figure S5: UV/visible absorption spectra in different spectral ranges and different concentrations of **1b** and **1c** in MeOH, and upon addition of pyridine; Figure S6: optimized geometries of **1a**, **1b**, **2a**, and **2b**.
