# Supplementary material for: Solid-State Nonlinear Optical Properties of Mononuclear Copper(II) Complexes with Chiral Tridentate and Tetradentate Schiff Base Ligands
Source: Materials (Basel). 2019 Nov 1;12(21):3595. doi: 10.3390/ma12213595 (PMC6862439; doi:10.3390/ma12213595)
Supplement: Supplementary file 1 [file materials-12-03595-s001.pdf]

materials

Solid-State Nonlinear Optical Properties of Mononuclear Copper(II) Complexes with Chiral Tridentate and Tetradentate Schiff Base Ligands

Luca Rigamonti [1,2,*], Alessandra Forni [2,3], Elena Cariati [2], Gianluca Malavasi [1], and Alessandro Pasini [2]

1 Dipartimento di Scienze Chimiche e Geologiche, Università degli Studi di Modena e Reggio Emilia, via G. Campi 103, 41125 Modena, Italy; gianluca.malavasi@unimore.it (G.M.)
2 Dipartimento di Chimica, Università degli Studi di Milano, via C. Golgi 19, 20133 Milano, Italy; elena.cariati@unimi.it (E.C.), alessandro.pasini@unimi.it (A.P.)
3 Istituto di Scienze e Tecnologie Molecolari, Consiglio Nazionale delle Ricerche, via C. Golgi 19, 20133 Milano, Italy alessandra.forni@istm.cnr.it (A.F.)
* Correspondence: luca.rigamonti@unimore.it or luca.rigamonti@yahoo.com (L.R.); Tel.: +39-059-205-8646

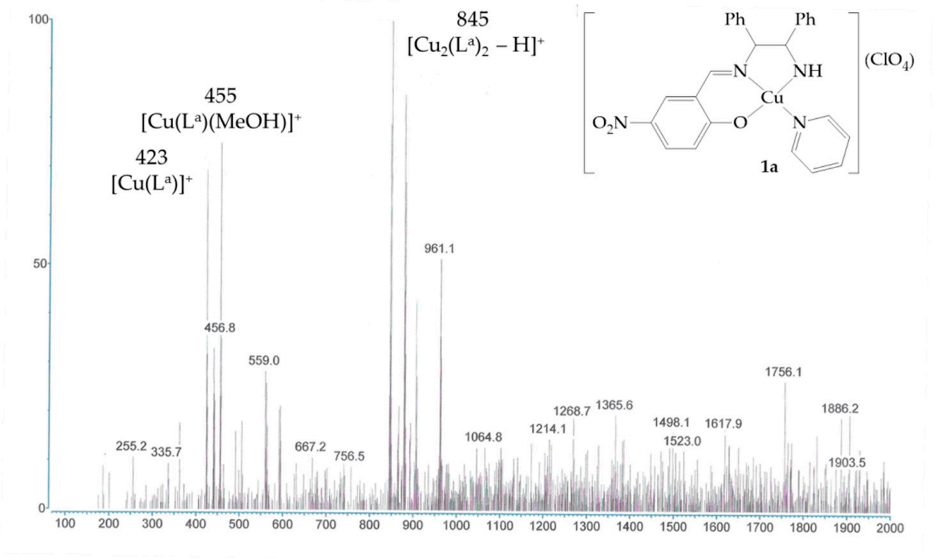
845
[Cu2(La)2 – H]+
455
[Cu(La)(MeOH)]+
423
[Cu(La)]+
Ph
Ph
N
NH
Cu
O2N
O
N
(ClO4)
1a
961.1
456.8
559.0
1756.1
1268.7
1365.6
1886.2
1498.1
1617.9
1214.1
1523.0
1064.8
255.2
335.7
667.2
756.5
1903.5

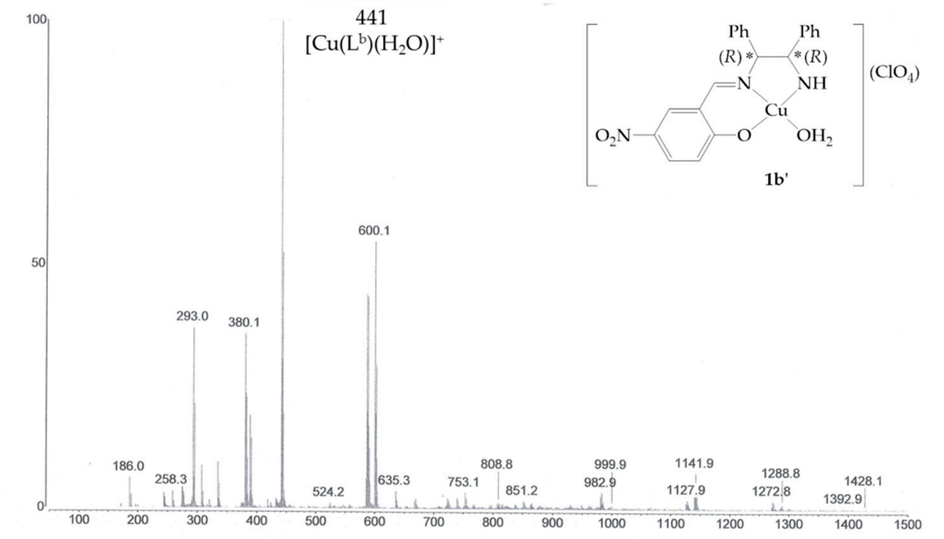
441
[Cu(Lb)(H2O)]+
Ph
Ph
(R)*
*(R)
N
NH
Cu
O2N
O
OH2
(ClO4)
1b'
600.1
293.0
380.1
186.0
258.3
524.2
635.3
753.1
808.8
851.2
999.9
982.9
1141.9
1127.9
1288.8
1272.8
1428.1
1392.9

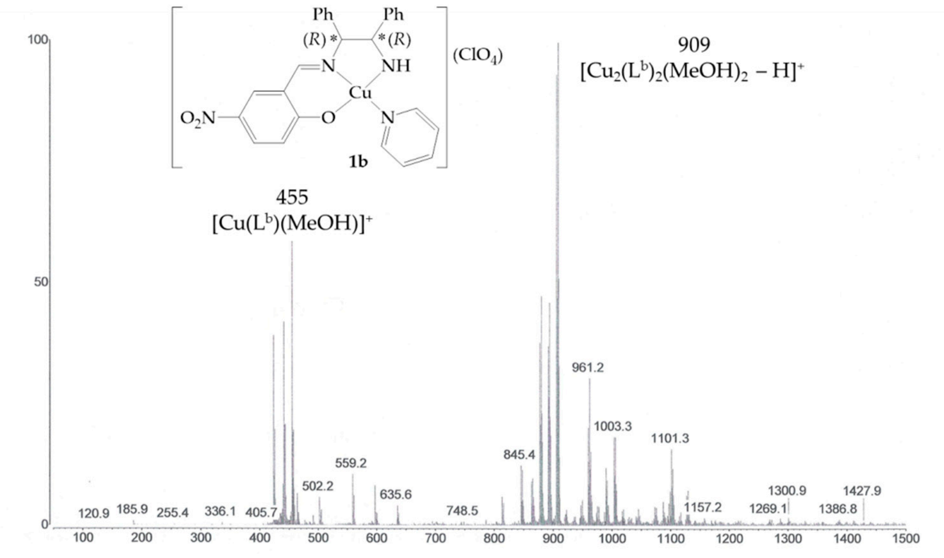
Ph
Ph
(R)*
*(R)
N
NH
Cu
O2N
O
N
(ClO4)
1b
909
[Cu2(Lb)2(MeOH)2 – H]+
455
[Cu(Lb)(MeOH)]+
961.2
1003.3
1101.3
845.4
559.2
502.2
635.6
748.5
120.9
185.9
255.4
336.1
405.7
1157.2
1269.1
1300.9
1386.8
1427.9

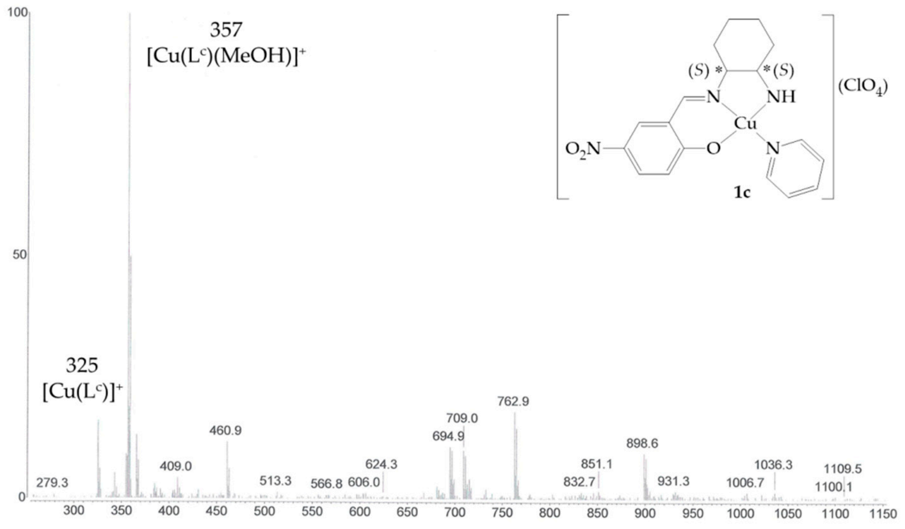
100
50
0
357
[Cu(L^c)(MeOH)]+
325
[Cu(L^c)]+
279.3
409.0
460.9
513.3
566.8
606.0
624.3
694.9
709.0
762.9
832.7
851.1
898.6
931.3
1006.7
1036.3
1100.1
1109.5
300 350 400 450 500 550 600 650 700 750 800 850 900 950 1000 1050 1100 1150
(S)
(S)
N
NH
Cu
O
N
O2N
(ClO4)
1c

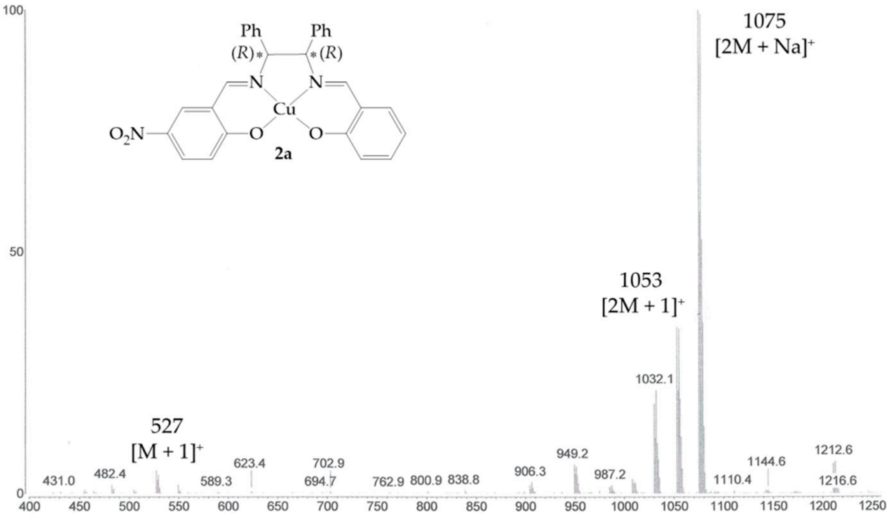
100
50
0
Ph
Ph
(R)
(R)
N
N
Cu
O
O
O2N
2a
1075
[2M + Na]+
1053
[2M + 1]+
1032.1
527
[M + 1]+
431.0
482.4
589.3
623.4
694.7
702.9
762.9
800.9
838.8
906.3
949.2
987.2
1110.4
1144.6
1212.6
1216.6
400 450 500 550 600 650 700 750 800 850 900 950 1000 1050 1100 1150 1200 1250

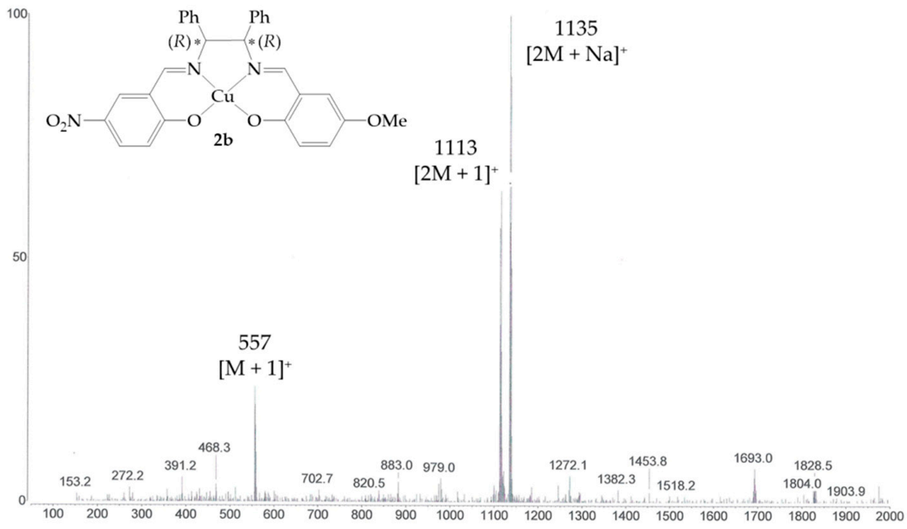

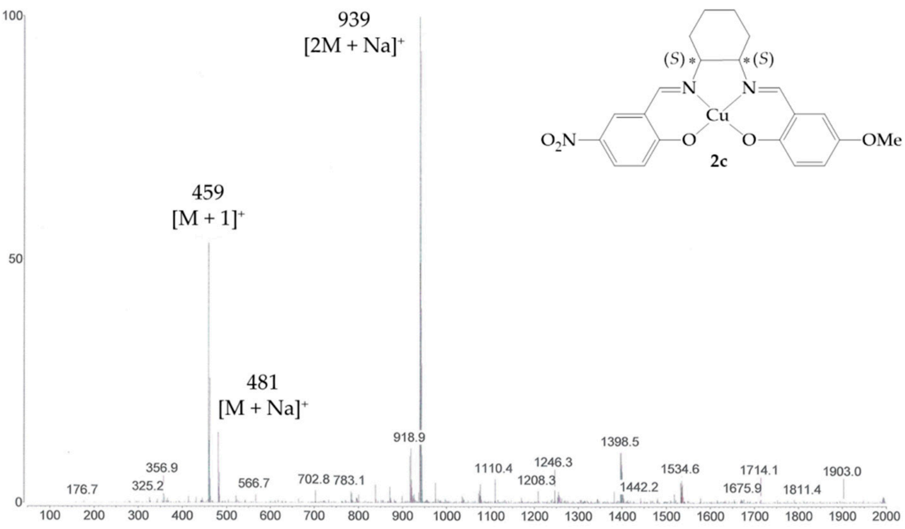

Figure S1. MS-ESI$^+$ spectra of compounds **1a–c**, **1b′** and **2a–c** registered in MeOH solution.

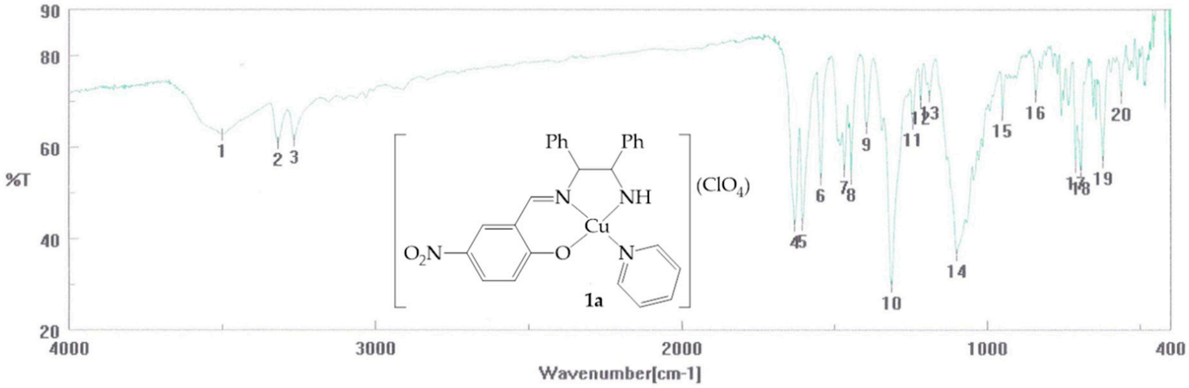
%T
Wavenumber[cm-1]
Ph
NH
Cu
O₂N
1a
(ClO4)

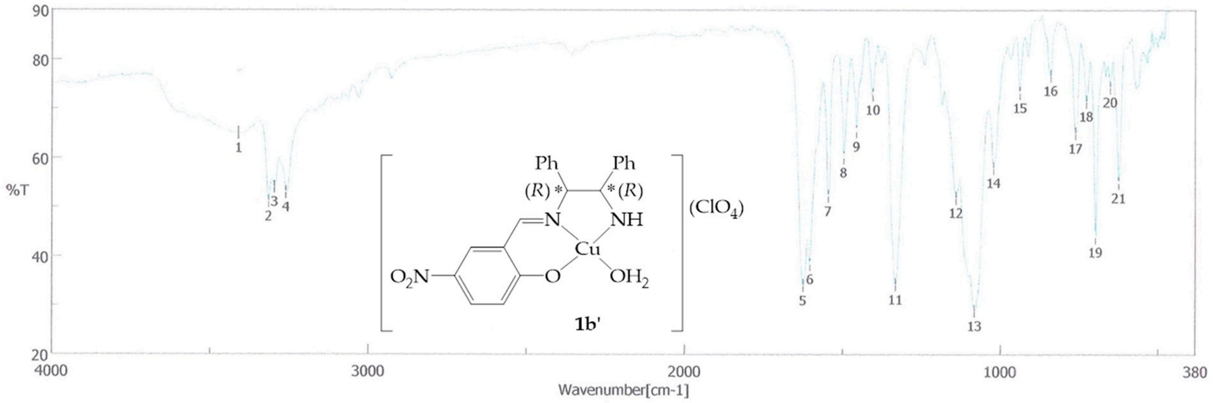
%T
Wavenumber[cm-1]
(R)
NH
Cu
OH2
O₂N
1b'
(ClO4)

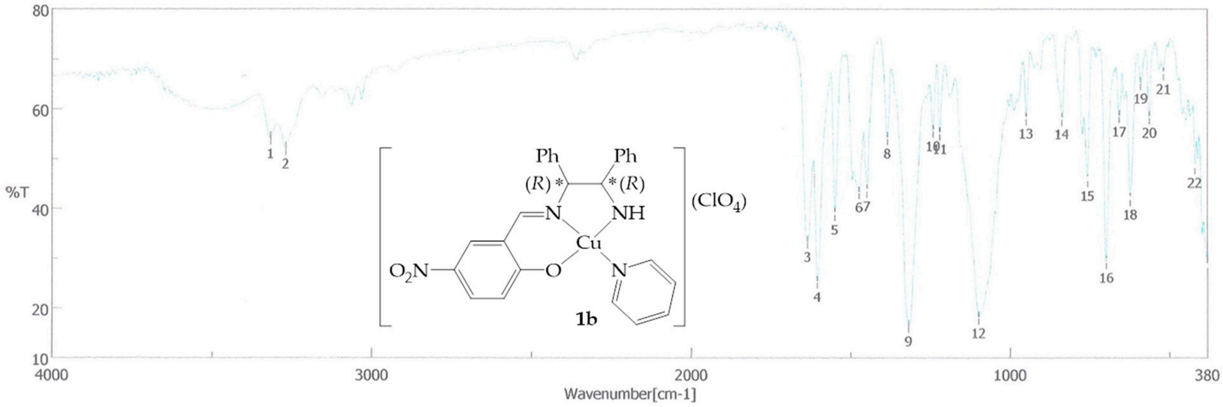
%T
Wavenumber[cm-1]
(R)
NH
Cu
O₂N
1b
(ClO4)

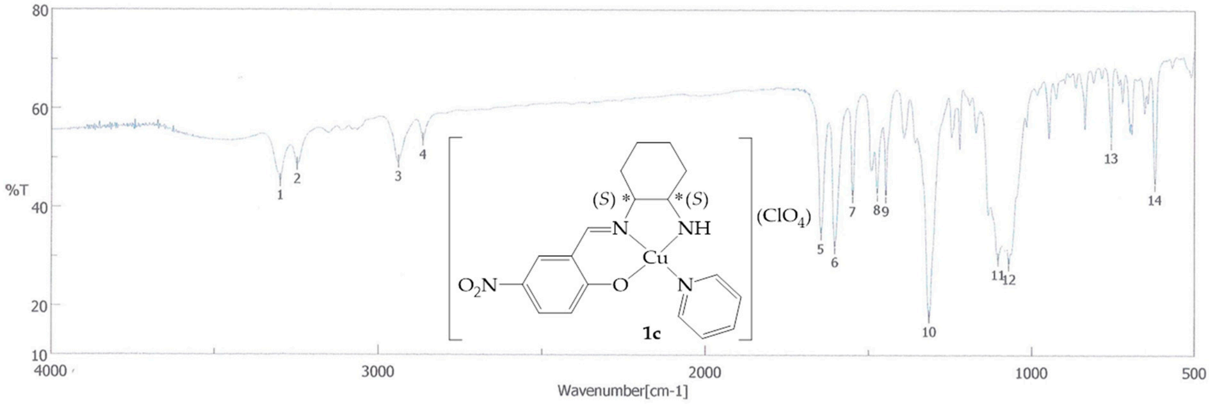
%T
Wavenumber[cm-1]
(S)
NH
Cu
O₂N
1c
(ClO4)

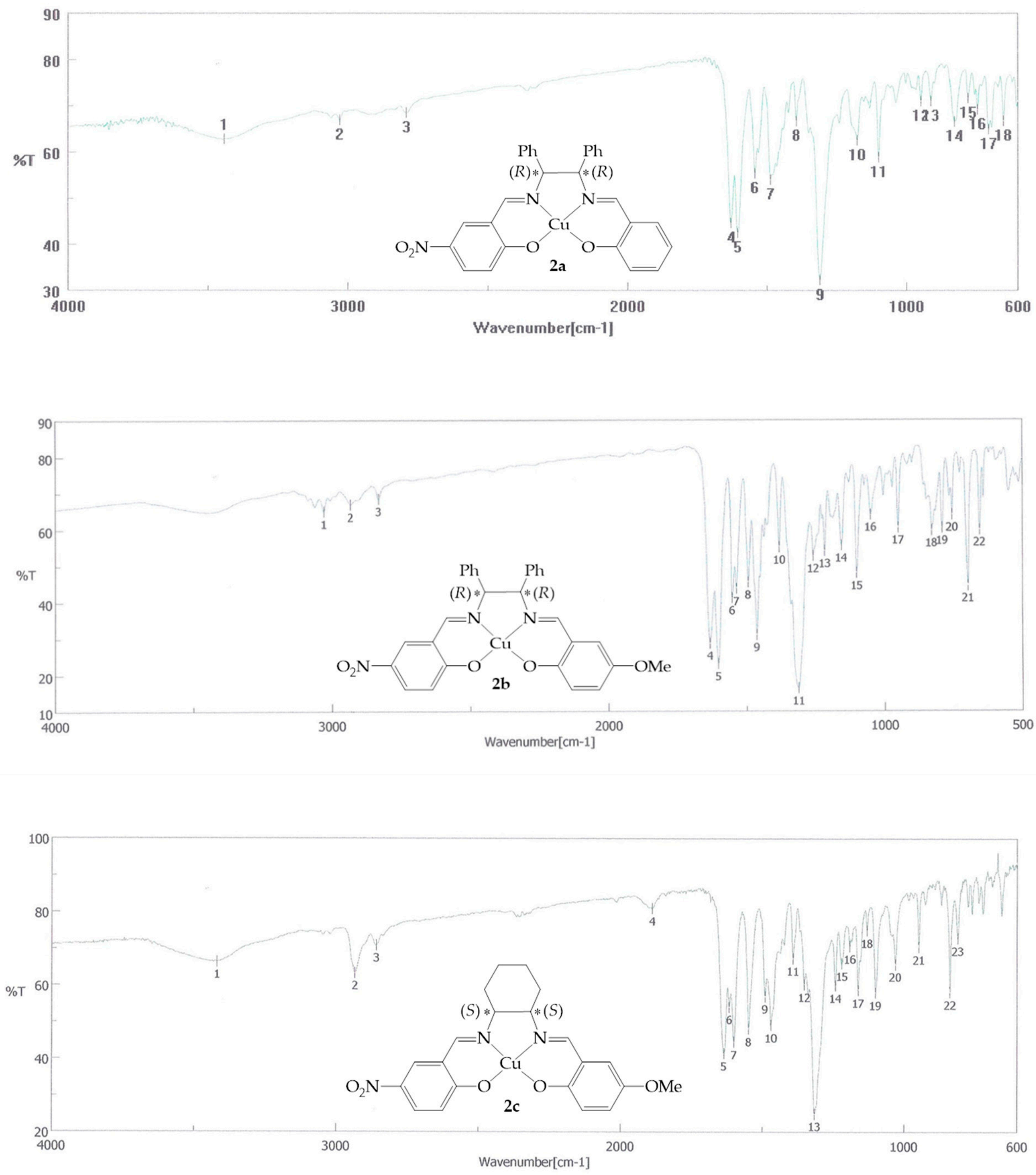

Figure S2. FT-IR spectra of compounds **1a–c**, **1b′** and **2a–c** registered as KBr disks.

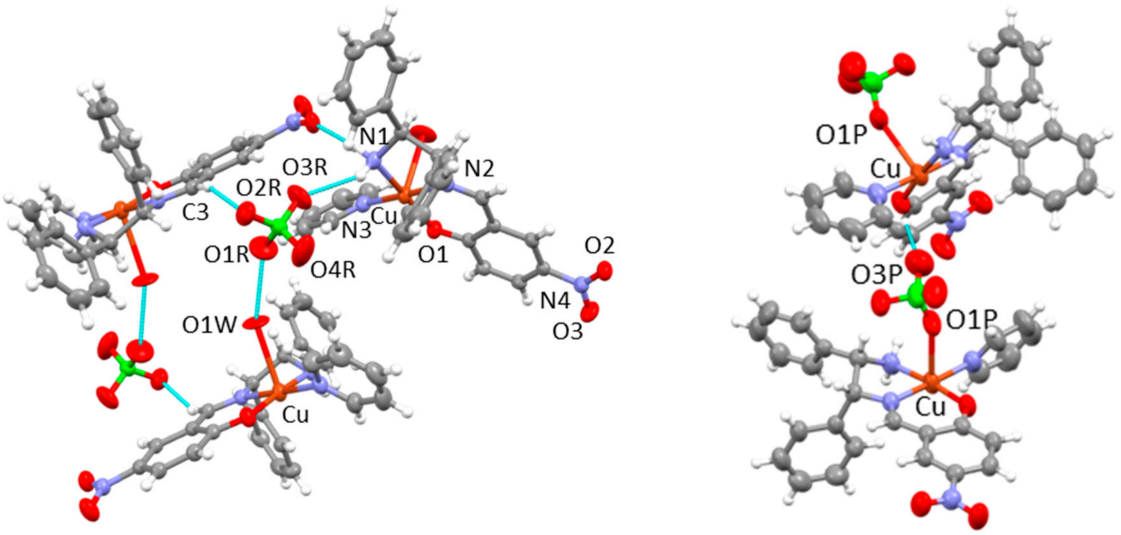

(**a**) (**b**)

Figure S3. Fragments of the X-ray crystal structure of **1a**·0.5H_2O with partial atom numbering scheme, showing in (**a**) the most populated perchlorate anion and the water molecule, while in (**b**) the minority perchlorate anion and the chain alignment. Displacement ellipsoids are drawn at 30% probability. Color code: Cu = orange, Cl = green, O = red, N = light blue, C = grey, H = white, intermolecular contacts are shown as cyan lines.

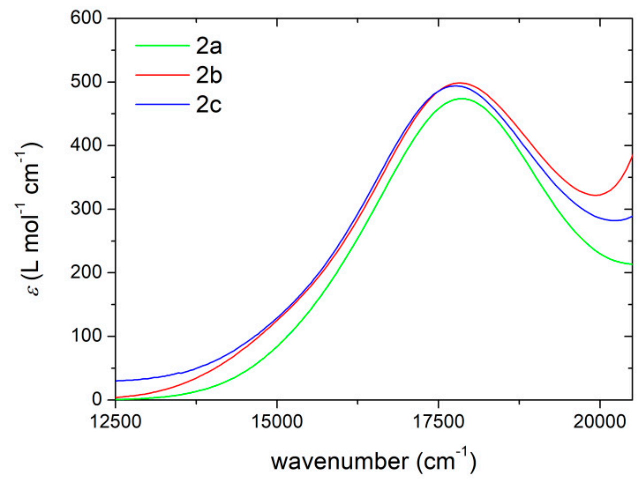

Figure S4. UV-visible absorption spectra in the 12500–20000 cm^{-1} range (*d*–*d* transition region) of **2a** and **2b** in 10^{-3} and **2c** in 5×10^{-4} mol L^{-1} $CHCl_3$ solutions.

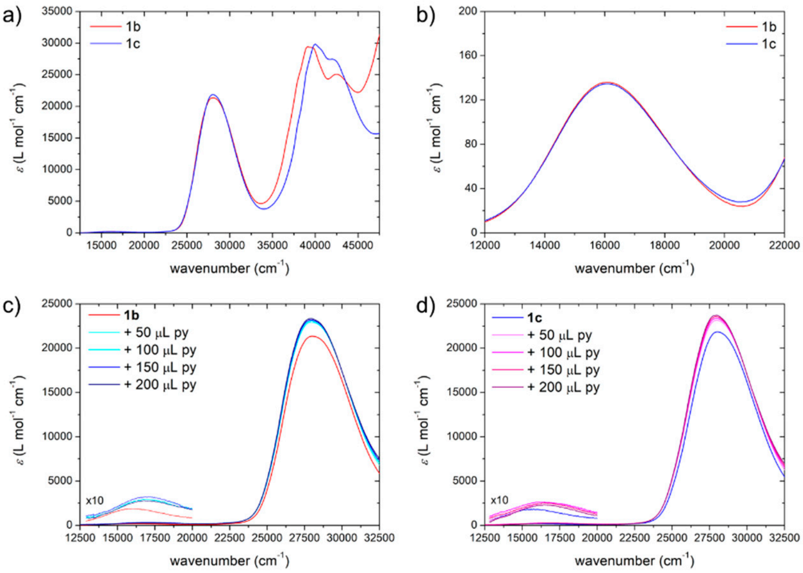

Figure S5. UV-visible absorption spectra in different spectral ranges of **1b** and **1c** in a) 4×10^{-5} mol L^{-1} MeOH solution, b) 10^{-3} mol L^{-1} MeOH solution (zoom over the 12500–20000 cm^{-1} range for the *d-d* transition region), c and d) 2 mL of the 4×10^{-5} mol L^{-1} MeOH solution of **1b** and **1c** upon subsequent addition of increasing amount of pyridine (data corrected for the occurring dilution).

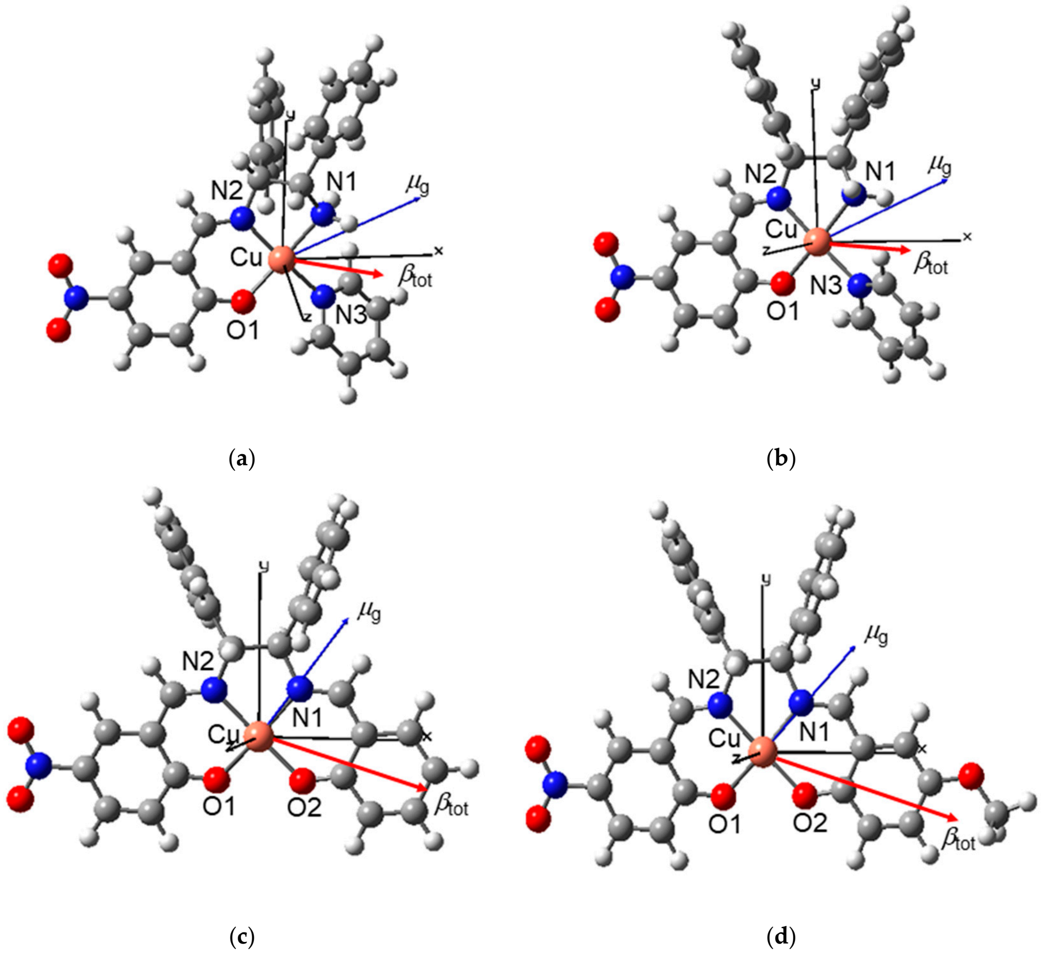

Figure S6. Optimized geometries with atom numbering of (a) **1a**, (b) **1b**, (c) **2a** and (d) **2b** with μ_g and β_{tot} vectors expressed in arbitrary units; color code: Cu = light orange, O = red, N = blue, C = grey, H = white.
